# Elevated Blood Pressure and Risk Factors in 19-Year-Olds in Serbia: A Cross-Sectional Study

**DOI:** 10.3390/medicina62010119

**Published:** 2026-01-06

**Authors:** Marija Sekulic, Milos Stepovic, Marija Sorak, Sara Mijailovic, Zlata Rajkovic Pavlovic, Maja Vulovic, Olivera Radmanovic, Branimir Radmanovic, Jelena Vuckovic Filipovic, Jagoda Gavrilovic, Bojana Jovanovic, Bojana Spasic, Nevena Folic, Vesna Rosic, Tode Dragicevic, Vladan Markovic

**Affiliations:** 1 Department of Hygiene and Ecology, Faculty of Medical Sciences, University of Kragujevac, 34000 Kragujevac, Serbia; msekulic82@gmail.com; 2 Department of Anatomy, Faculty of Medical Sciences, University of Kragujevac, 34000 Kragujevac, Serbia; maja@fmn.kg.ac.rs; 3 Department of Gynecology and Obstetrics, Faculty of Medical Sciences, University of Kragujevac, 34000 Kragujevac, Serbia; 4 Department of Medical Statistics and Informatics, Faculty of Medical Sciences, University of Kragujevac, 34000 Kragujevac, Serbia; saramijailovic212@gmail.com; 5 Department of Dentistry, Faculty of Medical Sciences, University of Kragujevac, 34000 Kragujevac, Serbia; zlatakg@yahoo.com; 6 Department of Internal Medicine, Faculty of Medical Sciences, University of Kragujevac, 34000 Kragujevac, Serbia; olja.radmanovic@gmail.com (O.R.); jelenavufi@gmail.com (J.V.F.); 7 Department of Psychiatry, Faculty of Medical Sciences, University of Kragujevac, 34000 Kragujevac, Serbia; biokg2005@yahoo.com; 8 Department of Infectious Diseases, Faculty of Medical Sciences, University of Kragujevac, 34000 Kragujevac, Serbia; jgavrilovic@outlook.com; 9 Health Care Service, Military Medical Academy, 11000 Belgrade, Serbia; bojanajovanovic14@gmail.com; 10 Faculty of Medical Sciences, University of Kragujevac, 34000 Kragujevac, Serbia; 11 Clinic for Physical Medicine and Rehabilitation, Military Medical Academy, 1100 Belgrade, Serbia; bokica.spasic@gmail.com; 12 Department of Pediatrics, Faculty of Medical Sciences, University of Kragujevac, 34000 Kragujevac, Serbia; nevena.folic@yahoo.com; 13 Department of Histology and Embriology, Faculty of Medical Sciences, University of Kragujevac, 34000 Kragujevac, Serbia; vesnanesic@yahoo.com; 14 Faculty of Medicine, University of Pristina, 38220 Kosovska Mitrovica, Serbia; dragicevic.tode@gmail.com; 15 Department of Radiology, Faculty of Medical Sciences, University of Kragujevac, 34000 Kragujevac, Serbia; drjack.vm@gmail.com

**Keywords:** young adult, hypertension, risk factors, health lifestyle

## Abstract

*Background and Objectives*: Hypertension in young adulthood is increasingly recognized as a precursor to future cardiovascular disease. Early identification of modifiable risk factors, such as dietary habits, lifestyle behaviors, and psychological indicators, is critical for prevention. This study aimed to examine the elevated clinic blood pressure and associated factors in 19-year-old individuals in Serbia. *Materials and Methods*: A cross-sectional study was conducted using data from the 2019 Fourth National Health Survey. A total of 212 participants underwent blood pressure measurement, anthropometric assessment, and completed standardized questionnaires on diet, physical activity, depressive symptoms, and sociodemographic characteristics. Blood pressure was classified according to ESC/ESH guidelines. Associations with elevated blood pressure were assessed using chi-square tests and multivariate logistic regression. *Results*: Most participants had optimal or normal blood pressure, while 18.9% had elevated blood pressure, including high-normal and Grade I–II hypertension. Elevated blood pressure was more prevalent among males and was associated with depressive symptoms. Nutrition status was significantly associated with elevated blood pressure, and some dietary habits like consumption of pure fruit or vegetable juices and the intake of processed meat products. Other socioeconomic factors, eating habits and physical activity were not significantly correlated. In multivariate logistic regression, elevated arterial blood pressure was significantly associated with consuming pure fruit or vegetable juices less than once per week (OR = 3.239; 95% CI: 1.413–7.427) and with consuming processed meat products several times per week in comparison to the daily consumption (OR = 0.325; 95% CI: 0.130–0.812), while no other variables remained statistically significant. *Conclusions*: Clinically elevated arterial blood pressure is present in a substantial proportion of 19-year-olds. Early lifestyle interventions targeting nutrition and psychological health may prevent progression to hypertension and reduce long-term cardiovascular risk.

## 1. Introduction

Hypertension, or elevated blood pressure, is a major preventable risk factor for cardiovascular morbidity and mortality, often developing silently over years without symptoms. It is estimated that globally, over 1.28 billion adults aged 30–79 years have hypertension, with a substantial proportion unaware of their condition [1]. Hypertension prevalence in young adults has been rising in parallel with obesity and unhealthy lifestyle behaviors, and early onset of elevated blood pressure significantly increases the risk of cardiovascular events later in life [2]. If untreated, hypertension accelerates vascular damage, contributes to atherosclerosis, left ventricular hypertrophy, and increases the likelihood of stroke, myocardial infarction, and chronic kidney disease [3].

The Australian, Canadian, European, and UK guidelines define hypertension as blood pressure ≥140/90 mmHg using standard clinic measurement methods. It is estimated that 37% of all hypertension in childhood is attributed to obesity [4].

Early-onset hypertension is an important risk factor for cardiovascular disease and stroke later in life [5]. Alarmingly, data from 2021–2023 show that 21.3% (20.4 million) of young adults aged 18–39 years had stage 1 or 2 hypertension, yet only 28.3% were aware of their condition and only 5.6% achieved blood pressure control to <130/80 mmHg [6]. This low awareness and control of hypertension highlights the importance of preventive strategies and early detection, particularly in populations at risk due to obesity or unhealthy lifestyle behaviors.

Obesity is one of the most common conditions that has a global burden on health care, causing severe consequences of morbidity and mortality worldwide [7,8]. It is defined as an abnormal accumulation of body fat over all organs and tissues, typically measured using Body Mass Index (BMI), while a BMI over 30 kg/m^2^ is classified as obesity [9]. This multisystem disorder is associated with five out of the ten leading causes of mortality and disability, including cardiovascular disease, type-2 diabetes, cancer, hypertension, and stroke [10]. Also, it increases the risk factor for metabolic syndrome associated with cardio-metabolic health conditions [11]. Despite all the global progress of the 21st century, the prevalence of obesity is increasing all over the world. According to the WHO, more than 100 million children are overweight, while childhood obesity is one of the most serious health challenges [12].

The period between 20 and 30 years, defined as young adulthood, represents a transition from adolescence and involves life changes related to education, work, and social relationships [13]. Starting independent life and self-care may be challenging, and when combined with health-endangering behaviors such as smoking, excessive alcohol consumption, poor diet, physical inactivity, and obesity, it significantly increases the risk for developing hypertension [14]. Young adults generally have a lower prevalence of chronic diseases compared to middle-aged and older adults; still, adolescence and early adulthood are critical periods for adopting healthy lifestyles that lay the foundation for future cardiovascular health [15]. This vulnerable period may lead to reduced physical activity and weight gain, both of which are recognized contributors to elevated blood pressure [16].

Results from over 100,000 participants in the US Health Professional Follow-Up Study and the Nurses’ Health Study showed that obesity in early adulthood is associated with an increased risk of major chronic diseases, including hypertension [17]. Adolescent obesity is also linked with a higher risk of premature death [18]. If obesity develops in young adults, compared to later in life, it accelerates the development of cardiovascular disease and promotes atherosclerotic changes in blood vessels over time. Long-term hypercholesterolemia during adulthood further contributes to atherosclerosis [12,19]. Atherosclerotic changes increase vascular resistance, contributing to elevated blood pressure.

The study focused specifically on individuals aged 19 years because this age represents a critical transitional period between adolescence and adulthood, during which lifestyle habits, dietary patterns, physical activity, and psychosocial factors become more established and may significantly influence long-term cardiovascular health. Early identification of risk factors for hypertension, including obesity, sedentary behavior, and dietary habits, at this stage allows for timely interventions that could prevent the progression of elevated blood pressure into adulthood. Additionally, focusing on a single age cohort minimizes variability related to developmental differences, providing a more homogeneous population for examining associations between hypertension and modifiable lifestyle and psychosocial factors. This approach ensures that observed relationships reflect true associations rather than age-related confounding, enhancing the validity and interpretability of the findings.

Given these trends, the study aims to investigate nutritional habits, lifestyle factors, and their relationship to hypertension development in young adults. Understanding these associations can help identify key risk factors and guide public health strategies and individual preventive measures to reduce early-onset hypertension and its long-term consequences on cardiovascular health.

## 2. Materials and Methods

### 2.1. Study Design

The research was designed as a cross-sectional study, conducted to assess elevated clinic blood pressure among young individuals aged 19 and to determine its association with nutrition levels, lifestyle factors, psychological indicators, and sociodemographic characteristics.

### 2.2. Target Population

The target population consisted of individuals aged 19 years residing in the territory of the Republic of Serbia, excluding the Autonomous Province of Kosovo and Metohija. Data were obtained from the Fourth National Health Survey of the Population of Serbia (2019), implemented by the Ministry of Health of the Republic of Serbia, the Institute of Public Health of Serbia “Dr Milan Jovanović Batut,” and the Statistical Office of the Republic of Serbia.

The study was conducted in accordance with the methodology and instruments of the European Health Interview Survey (EHIS—wave 3).

The study included respondents who were exactly 19 years old and who provided informed consent for participation, while excluding individuals younger or older than 19 years, those who were physically or mentally unable to participate, and respondents who did not provide informed consent.

### 2.3. Sampling

A stratified two-stage sampling method was applied to ensure representativeness according to the type of settlement (urban/rural) and geographical regions (Belgrade, Vojvodina, Šumadija and Western Serbia, Southern and Eastern Serbia). The sampling frame was based on the 2011 Population Census of the Republic of Serbia.

For the subgroup of participants aged 19 years, the minimum sample size required to estimate the prevalence of arterial hypertension of 3.4% was calculated with a 95% confidence level, an absolute precision of ±3%, a design effect of 1.5, and an expected response rate of 80%. The calculation was performed using the standard approach for estimating a population proportion, applying the formula, where Z represents the standard normal deviate for a 95% confidence level (1.96), p is the expected prevalence, d is the desired precision, and DEFF denotes the design effect. The result indicated that a minimum of 210 valid respondents is required for this subgroup, which falls within the total study sample of 13,000 participants and ensures representativeness for the population of 19-year-olds in Serbia [20,21].

### 2.4. Instruments and Variables

The study used standardized questionnaires and measurements developed in accordance with the EHIS wave 3 protocol, adapted to the objectives of this research. Data were collected through: Face-to-face interviews conducted by trained interviewers, Self-administered questionnaires, and Anthropometric and blood pressure measurements.

### 2.5. Variables

Arterial hypertension: In adults (≥18 years), arterial blood pressure is classified according to the European Society of Cardiology (ESC) and European Society of Hypertension (ESH) guidelines as follows: optimal blood pressure is defined as systolic <120 mmHg and diastolic <80 mmHg, normal as 120–129/80–84 mmHg, and high-normal as 130–139/85–89 mmHg. Hypertension is diagnosed when systolic blood pressure is ≥140 mmHg and/or diastolic blood pressure is ≥90 mmHg, confirmed by at least two measurements on two or more separate occasions, and is further categorized into grade 1 (140–159/90–99 mmHg, mild), grade 2 (160–179/100–109 mmHg, moderate), and grade 3 (≥180/≥110 mmHg, severe) [22]. In accordance with ESC/ESH recommendations, we acknowledge that a clinical diagnosis of hypertension requires measurements on separate visits; therefore, our outcome reflects elevated clinic blood pressure measured at a single survey visit, rather than confirmed hypertension prevalence.

The independent variables in this study included obesity, measured using body mass index (BMI = kg/m^2^) calculated from participants’ height and weight, and categorized as underweight (BMI < 18.5), normal weight (18.5–24.9), overweight (25–29.9), and obese (≥30); dietary habits, assessed based on the frequency of consumption of fruits, vegetables, sweets, fast food, and carbonated drinks; physical activity, evaluated using the EHIS-PAQ (Physical Activity Questionnaire) [23],.covering activity during leisure time, work, and transport (walking and cycling); sedentary behavior, measured as the average daily time spent sitting; self-perceived health, reflecting participants’ subjective assessment of their health status (excellent, good, fair, poor, very poor); depressive symptoms, measured by Patient Health Questionnaire (PHQ-8), a brief questionnaire used to assess the presence and severity of depressive symptoms in the general population. It measures depressive symptoms based on eight questions referring to experiences during the previous two weeks. The total score is calculated by summing the item responses and is categorized according to symptom severity, such as no symptoms, mild, or severe depressive symptoms. The PHQ-8 is an internationally standardized instrument and is widely used in population-based surveys for national health research. In our study, we applied this instrument in line with the methodology of the European Health Interview Survey (EHIS), which further supports its validity and comparability with international data [24]; attitudes toward risk behaviors, capturing participants’ perceptions of how smoking, alcohol, and drug use affect health; and sociodemographic characteristics, including gender, type of settlement (urban/rural), region, level of education, economic status, and household composition.

#### Measurement Procedures

1. Blood Pressure: measured using a digital sphygmomanometer (Ri Champion N) with appropriate cuff sizes. At least two measurements were taken for each participant, and the average value was used for analysis.
2. Body Weight and Height: measured according to standardized procedures using a calibrated electronic medical scale and an adjustable stadiometer (SECA). BMI: calculated as weight divided by height squared (kg/m^2^).

### 2.6. Ethical Considerations

The study was conducted in accordance with the Declaration of Helsinki, the Law on Personal Data Protection of the Republic of Serbia, and the General Data Protection Regulation (GDPR). All participants provided written informed consent prior to participation. The database was used with the permission of the Institute of Public Health of Serbia “Dr Milan Jovanović Batut” and was officially provided to the University of Kragujevac for research purposes.

### 2.7. Statistical Tests

Statistical data analysis was performed using IBM SPSS Statistics for Windows, Version 22.0 (IBM Corp., Armonk, NY, USA). Numerical variables were categorized into appropriate groups to meet the analytical requirements. The chi-square test was used to assess associations between categorical variables, while Fisher’s exact test was applied in cases of small expected frequencies. To identify factors associated with elevated arterial blood pressure, a binary logistic regression analysis was conducted. Statistical significance was set at a probability level of *p* < 0.05. The results are presented in both tabular and textual form.

## 3. Results

### 3.1. Sociodemographic Characteristics According to Blood Pressure Categories

A total of 212 participants aged 19 years were included in the study, of whom 129 (60.8%) were male and 83 (39.2%) were female. According to the classification of arterial blood pressure, 72 participants (34.0%) had optimal values, 100 (47.2%) had normal values, while high-normal blood pressure was recorded in 31 participants (14.6%). Grade I hypertension was present in 7 participants (3.3%), and Grade II hypertension in 2 participants (0.9%).

Blood pressure values were further grouped into two categories: optimal and normal blood pressure in the first group (172 participants, 81.1%), and high-normal blood pressure together with Grade I and Grade II hypertension in the second group (40 participants, 18.9%). In the group of participants with elevated blood pressure, a higher proportion of males was observed—75.0% (30)—compared to the group with normal blood pressure, where males accounted for 57.6% (99) (*p* = 0.042). Other sociodemographic characteristics, such as region, household size, wealth index, and employment status, did not differ between the groups (*p* > 0.05) (Table 1).

### 3.2. Health-Related Characteristics According to Blood Pressure Categories

Self-rated general health did not differ between participants with normal and elevated arterial blood pressure (*p* = 0.098). Symptoms of depression showed a statistically significant association with blood pressure level (*p* = 0.002). In the group of participants with elevated blood pressure, mild symptoms were recorded in 97.5% (34) and moderate to severe symptoms in 2.5% (1). In the group with normal blood pressure, 77.9% (138) of participants reported depressive symptoms, of which 71.5% (123) had mild and 6.4% (11) had moderate to severe symptoms. Physical activity parameters, including walking and time spent sitting during the day, did not differ significantly between the groups (*p* > 0.05) (Table 2).

### 3.3. Dietary Habits According to Blood Pressure Categories

Regarding dietary habits, statistically significant differences were observed in the frequency of consuming pure fruit or vegetable juices, sugary soft drinks, and processed meat products (*p* < 0.05), while the remaining dietary habits were not associated with blood pressure categories (Table 3). Pure fruit or vegetable juices were consumed less than once per week by 42.5% (17) of participants with elevated blood pressure, compared to 23.3% (40) of those with normal blood pressure. Sugary soft drinks were consumed daily by 25.0% (10) of participants with elevated blood pressure, versus 9.9% (17) of participants with normal blood pressure. Processed meat products were consumed daily by 37.5% (15) of participants with elevated blood pressure, compared to 12.2% (21) in the normal blood pressure group.

The average BMI value was 22.60 ± 3.74. Participants with elevated blood pressure had a significantly higher mean BMI compared to those with normal blood pressure (23.97 ± 3.09 vs. 22.27 ± 3.82, *p* = 0.010). According to nutritional status categories, 27 participants (13.2%) were underweight, 136 (64.2%) had normal weight, 36 (17.0%) were classified as overweight, while obesity was recorded in 12 participants (5.7%). As shown in Table 4, nutritional status demonstrated a statistically significant association with arterial blood pressure categories (*p* = 0.048). In the group of participants with elevated blood pressure, overweight (27.5%, n = 11) and obesity (7.5%, n = 3) were more common compared to the group with normal blood pressure, where these categories accounted for 14.5% (n = 25) and 5.2% (n = 9), respectively.

### 3.4. Predictors of Elevated Blood Pressure

To assess the factors associated with elevated arterial blood pressure, a binary logistic regression analysis was conducted. The model included variables that showed statistical significance: sex, frequency of consuming pure fruit or vegetable juices, sugar-sweetened non-alcoholic beverages, processed meat products, as well as nutritional status categories. The variable “depressive symptoms” was not included in the model because, in the group with elevated blood pressure, there were no participants without depressive symptoms; therefore, the variable was not suitable for analysis.

Due to the small number of participants in certain categories, the original variables were collapsed to ensure model stability. The frequency of consuming pure fruit or vegetable juices was grouped into two categories: frequent consumption (4–6 times per week and 1–3 times per week) and infrequent consumption (less than once per week and never).

The frequency of consuming sugar-sweetened non-alcoholic beverages and processed meat products was grouped into three categories: multiple times per day, multiple times per week (4–6 times and 1–3 times per week), and less than once per week or never.

Nutritional status categories were condensed into two groups: underweight and normal weight combined into one category, and overweight and obesity combined into the second category.

The final model was statistically significant (χ^2^ = 28.972, *p* < 0.001), with satisfactory fit according to the Hosmer–Lemeshow test (*p* = 0.832). Results of the multivariate logistic regression showed that the frequency of consuming pure fruit or vegetable juices was a significant predictor of elevated arterial blood pressure. Participants who consumed these juices less than once per week had a higher likelihood of elevated blood pressure compared to those who consumed them frequently (OR = 3.239; 95% CI: 1.413–7.427; *p* = 0.005).

Additionally, the frequency of consuming processed meat products was also significant. Participants who consumed these products multiple times per week had a lower likelihood of elevated arterial blood pressure compared to the reference group that consumed them daily (OR = 0.325; 95% CI: 0.130–0.812; *p* = 0.016). Sex, frequency of consuming sugar-sweetened beverages, and nutritional status categories did not emerge as independent predictors of elevated arterial blood pressure in the multivariate model (*p* > 0.05) (Table 5).

## 4. Discussion

Among 19-year-old participants, the clinically manifest hypertension was low, with elevated arterial blood pressure occurring more frequently in males and being associated with depressive symptoms and nutritional status, while most sociodemographic and lifestyle factors showed no significant differences. Dietary patterns emerged as the most relevant predictors, as infrequent consumption of pure fruit or vegetable juices was independently associated with elevated blood pressure, underscoring the importance of early dietary interventions to prevent hypertension in young adults.

In this study, 19-year-old participants showed a relatively low occurrence of elevated clinic blood pressure, with stage I hypertension present in 3.3% and stage II hypertension in 0.9% of participants. The majority had optimal or normal blood pressure values, which aligns with previous studies in similar populations of young adults [25,26]. However, a notable proportion of participants had high-normal blood pressure (14.6%), which may indicate an early stage of developing risk factors for hypertension, considering the well-known progression of elevated blood pressure during adolescence and early adulthood [26].

Male sex was identified as a significant factor associated with elevated blood pressure, consistent with literature reporting a higher prevalence of hypertension in young men compared to their female peers [27,28]. Other sociodemographic characteristics, including region of residence, household size, wealth index, and employment status, did not show a statistically significant association with elevated blood pressure, suggesting that in young adults, biological and lifestyle factors play a more prominent role than socioeconomic factors [28,29].

Interestingly, self-perceived general health did not differ according to blood pressure level, whereas depressive symptoms were significantly more frequent among participants with elevated blood pressure. Due to the limited number of participants, this association could not be confirmed in multivariable analyses. Similar associations between depressive symptoms and hypertension have been documented previously, indicating a possible bidirectional relationship between psychological status and blood pressure regulation in young adults [30,31,32].

Physical activity, including walking, total aerobic activity, and sedentary time, did not show a significant association with blood pressure, which could be due to relatively homogeneous activity levels within this age group or limitations of self-reported activity measures [33]. In contrast, diet emerged as a significant factor. Higher consumption of sweetened soft drinks, processed meat, and lower intake of pure fruit and vegetable juices were more common among participants with elevated blood pressure, consistent with previous studies linking unhealthy dietary habits to elevated blood pressure in young adults [34,35,36].

Nutritional status also showed a statistically significant association with blood pressure categories. Overweight and obesity were more prevalent among participants with elevated blood pressure, confirming the well-established impact of increased body weight on blood pressure regulation, even in young adult populations [37,38,39,40]. These findings highlight the need for preventive strategies that focus on lifestyle modifications, particularly diet and maintaining a healthy body weight, to reduce the risk of developing hypertension early in adulthood [41].

These findings are in line with international studies and provide a basis for targeted preventive policies aimed at early detection and intervention in the young adult population [42,43].

The strengths of this study include a homogeneous sample of 19-year-old participants, standardized blood pressure measurements, and a comprehensive evaluation of sociodemographic, psychological, nutritional, and dietary factors. The application of multivariate logistic regression allowed for the identification of independent associations, while the focus on modifiable lifestyle behaviors enhances the relevance of the findings for early prevention strategies.

This study is limited by its cross-sectional design and focus on a single age cohort, the use of single-visit clinic blood pressure measurements without ambulatory monitoring or repeated visits, reliance on self-reported dietary intake and physical activity with potential recall and social desirability bias, and a relatively small sample size in certain dietary categories, which may have resulted in imprecise estimates and unexpected associations. Focusing solely on 19-year-olds limits generalizability, and the small number of participants with clinically manifest hypertension, which was expected, restricting analysis of less common risk factors.

## 5. Conclusions

In this study of 19-year-old participants, most had optimal or normal arterial blood pressure, with a low elevated clinic blood pressure (Grade I: 3.3%; Grade II: 0.9%). Elevated blood pressure was more common among males and was associated with depressive symptoms, while other sociodemographic factors, self-rated health, and physical activity did not differ significantly between groups. Nutrition status showed a significant correlation as well. Dietary habits showed important associations: infrequent consumption of pure fruit or vegetable juices was a significant predictor of elevated blood pressure in both univariate and multivariate analyses, and any intake of processed meat products that was not daily consumption was associated with lower odds of elevated blood pressure in the multivariate model; however, this finding reflects a relative comparison between consumption categories and should not be interpreted as evidence of a protective or beneficial effect of processed meat intake. Sex, sugar-sweetened beverage intake, and nutritional status were not independent predictors in the final model. These findings highlight the importance of promoting healthy dietary habits, particularly fruit and vegetable intake, and early lifestyle interventions to prevent the development of elevated blood pressure in young adults.

## Figures and Tables

**Table 1:** Sociodemographic characteristics according to arterial blood pressure categories.

Variables	Total (n = 212)	Normal BP (n = 172)	Elevated BP (n = 40)	*p* *
*Region*				0.288
Vojvodina	51 (24.1%)	39 (22.7%)	12 (30.0%)	
Šumadija & Western Serbia	65 (30.7%)	53 (30.8%)	12 (30.0%)	
Southern & Eastern Serbia	47 (22.2%)	36 (20.9%)	11 (27.5%)	
Belgrade	49 (23.1%)	44 (25.6%)	5 (12.5%)	
*Sex*				0.042 *
Male	129 (60.8%)	99 (57.6%)	30 (75.0%)	
Female	83 (39.2%)	73 (42.4%)	10 (25.0%)	
*Household size*				0.122
1–3 members	47 (22.2%)	36 (20.9%)	11 (27.5%)	
4–5 members	105 (49.5%)	91 (52.9%)	14 (35.0%)	
≥6 members	60 (28.3%)	45 (26.2%)	15 (37.5%)	
*Wealth index*				0.431
Poorest & poor	97 (45.8%)	77 (44.8%)	20 (50.0%)	
Middle class	44 (20.8%)	34 (19.8%)	10 (25.0%)	
Rich & richest	71 (33.5%)	61 (35.5%)	10 (25.0%)	
*Employment status*				0.865
Unemployed	58 (27.5%)	46 (26.7%)	12 (30.8%)	
Inactive	121 (57.3%)	100 (58.1%)	21 (53.8%)	
Employed	32 (15.2%)	26 (15.1%)	6 (15.4%)	

* Chi-square test, *p* value less than 0.05.

**Table 2:** Self-rated health, depressive symptoms, and physical activity according to blood pressure categories.

Variable	Total (n = 212)	Normal BP (n = 172)	Elevated BP (n = 40)	*p* *
*Self-rated health*				0.098
Poor or very poor	1 (0.6%)	0 (0%)	1 (2.5%)	
Average	6 (3.4%)	6 (4.5%)	0 (0.0%)	
Good or very good	167 (96.0%)	128 (95.5%)	39 (97.5%)	
*Depressive symptoms*				0.002 *
No symptoms	38 (17.9%)	38 (22.1%)	0 (0.0%)	
Mild symptoms	162 (76.4%)	123 (71.5%)	34 (97.5%)	
Moderate to severe symptoms	12 (5.7%)	11 (6.4%)	1 (2.5%)	
*Walking*				0.174
≤150 min	182 (86.3%)	151 (87.8%)	31 (79.5%)	
>150 min	29 (13.7%)	21 (12.2%)	8 (20.5%)	
*Time spent sitting (typical day)*				0.739
≤6 h/day	125 (76.2%)	94 (75.2%)	31 (79.5%)	
>6 h/day	39 (23.8%)	31 (24.8%)	8 (20.5%)	

* Chi-square test, *p* value less than 0.05.

**Table 3:** Dietary habits according to arterial blood pressure categories.

Variable	Total (n = 212)	Normal BP (n = 172)	Elevated BP (n = 40)	*p* *
*Breakfast frequency*				0.735
Every day	154 (88.5%)	118 (88.1%)	36 (90.0%)	
Sometimes	18 (10.3%)	14 (10.4%)	4 (10.0%)	
Never	2 (1.1%)	2 (1.5%)	0 (0.0%)	
*Bread consumption*				0.392
Every day	146 (83.9%)	111 (82.8%)	35 (87.5%)	
Sometimes	22 (12.6%)	17 (12.7%)	5 (12.5%)	
Never	6 (3.4%)	6 (4.5%)	0 (0.0%)	
*Milk/dairy consumption*				0.832
Once or more per day	80 (46.0%)	60 (44.8%)	20 (50.0%)	
4–6 times/week	58 (33.3%)	46 (34.3%)	12 (30.0%)	
1–3 times/week	36 (20.7%)	28 (20.9%)	8 (20.0%)	
*Fresh fruit consumption*				0.859
Once or more per day	60 (28.3%)	50 (29.1%)	10 (25.0%)	
4–6 times/week	53 (25.0%)	43 (25.0%)	10 (25.0%)	
1–3 times/week	99 (46.7%)	79 (45.9%)	20 (50.0%)	
*Vegetable/salad consumption*				0.444
Once or more per day	72 (34.0%)	55 (32.0%)	17 (42.5%)	
4–6 times/week	63 (29.7%)	53 (30.8%)	10 (25.0%)	
1–3 times/week	77 (36.3%)	64 (37.2%)	13 (32.5%)	
*Pure fruit/vegetable juice consumption*				0.003 *
4–6 times/week	22 (10.4%)	20 (11.6%)	2 (5.0%)	
1–3 times/week	97 (45.8%)	87 (50.6%)	10 (25.0%)	
Less than once/week	57 (26.9%)	40 (23.3%)	17 (42.5%)	
Never	36 (17.0%)	25 (14.5%)	11 (27.5%)	
*Sugary soft drink consumption*				0.013 *
Once or more per day	27 (12.7%)	17 (9.9%)	10 (25.0%)	
4–6 times/week	38 (17.9%)	29 (16.9%)	9 (22.5%)	
1–3 times/week	90 (42.5%)	78 (45.3%)	12 (30.0%)	
Less than once/week	32 (15.1%)	14 (8.1%)	8 (20.0%)	
Never	25 (11.8%)	24 (14.0%)	1 (2.5%)	
*Red meat consumption*				0.486
Once or more per day	6 (2.8%)	4 (2.3%)	2 (5.0%)	
4–6 times/week	52 (24.5%)	40 (23.3%)	12 (30.0%)	
1–3 times/week	142 (67.0%)	117 (68.0%)	25 (62.5%)	
Less than once/week	12 (5.7%)	11 (6.4%)	1 (2.5%)	
*White meat consumption*				0.575
Once or more per day	8 (3.8%)	6 (3.5%)	2 (5.0%)	
4–6 times/week	52 (24.5%)	40 (23.3%)	12 (30.0%)	
1–3 times/week	152 (71.7%)	126 (73.3%)	26 (65.0%)	
*Fish/seafood consumption*				0.153
4–6 times/week	14 (6.6%)	12 (7.0%)	2 (5.0%)	
1–3 times/week	100 (47.2%)	87 (50.0%)	13 (32.5%)	
Less than once/week	86 (40.6%)	64 (37.2%)	22 (55.0%)	
Never	12 (5.7%)	9 (5.2%)	3 (7.5%)	
*Processed meat products*				0.003 *
Once or more per day	36 (17.0%)	21 (12.2%)	15 (37.5%)	
4–6 times/week	58 (27.4%)	48 (27.9%)	10 (25.0%)	
1–3 times/week	101 (47.6%)	88 (51.2%)	13 (32.5%)	
Less than once/week	10 (4.7%)	8 (4.7%)	2 (5.0%)	
Never	7 (3.3%)	7 (4.1%)	0 (0.0%)	
*Adding salt after food preparation*				0.174
Always, before tasting	18 (10.3%)	13 (9.7%)	5 (12.5%)	
Often, after tasting	32 (18.4%)	21 (15.7%)	11 (27.5%)	
Never/Rarely	124 (71.3%)	100 (74.6%)	24 (60.0%)	

* Chi-square test, *p* value less than 0.05.

**Table 4:** Nutritional status categories according to arterial blood pressure categories.

Nutritional Status	Total (n = 212)	Normal Blood Pressure (n = 172)	Elevated Blood Pressure (n = 40)	*p* *
Underweight	28 (13.2%)	27 (15.7%)	1 (2.5%)	0.048 *
Normal weight	136 (64.2%)	111 (64.5%)	25 (62.5%)	
Overweight	36 (17.0%)	25 (14.5%)	11 (27.5%)	
Obesity	12 (5.7%)	9 (5.2%)	3 (7.5%)	

* Chi-square test, *p* value less than 0.05.

**Table 5:** Results of univariate and multivariate logistic regression for elevated arterial blood pressure.

Variables		Univariate Logistic Regression		Multivariate Logistic Regression	
		OR (95% CI)	*p*	OR (95% CI)	*p*
*Sex*	Ref. Male				
	Female	0.452 (0.208–0.983)	0.045 *	0.568 (0.248–1.302)	0.181
*Frequency of consuming pure fruit or vegetable juices*	Reference: Several times per week				
	Less than once per week	3.841 (1.827–8.076)	0.000 *	3.239 (1.413–7.427)	0.005 *
*Frequency of consuming sugar-sweetened beverages*	Reference: Several times per day				
	Several times per week	0.334 (0.134–0.829)	0.018 *	0.971 (0.317–2.974)	0.959
	Less than once per week	0.319 (0.111–0.917)	0.034 *	0.649 (0.191–2.200)	0.487
*Frequency of consuming processed meat products*	Reference: Several times per day				
	Several times per week	0.237 (0.107–0.525)	0.000 *	0.325 (0.130–0.812)	0.016 *
	Less than once per week	0.187 (0.037–0.941)	0.042 *	0.254 (0.045–1.427)	0.120
*Nutritional status*	Reference: Underweight and normal weight				
	Overweight and obesity	2.186 (1.032–4.628)	0.041 *	2.157 (0.933–4.988)	0.072

* *p* value less than 0.05 (indicates where statistical significance was found).

## Data Availability

Data are unavailable due to privacy or ethical restrictions because the current owner of the rights, the Institute of Public Health of Serbia, “Milan Jovanović Batut” and the database was handed over to the University of Kragujevac with an official letter for the purpose of further research.
